# Correction: Nguyen T.L., et al. Role of Prokineticin Receptor-1 in Epicardial Progenitor Cells. *J. Dev. Biol.* 2013, *1*, 20–31

**DOI:** 10.3390/jdb8040032

**Published:** 2020-12-11

**Authors:** Thu Lan Nguyen, Adeline Gasser, Canan G. Nebigil

**Affiliations:** 1 Centre National de la Recherche Scientifique (CNRS), Université de Strasbourg, UMR7242, Ecole Supérieure de Biotechnologie de Strasbourg, F-67412 Illkirch, France; tleinnguyen@gmail.com (T.L.N.); gasseradeline@gmail.com (A.G.); 2 Center of Research of Biomedicine in Strasbourg (CRBS), Regenerative Nanomedicine, UMR1260, INSERM, University of Strasbourg, 1 rue Eugéne Boeckel, 67084 Strasbourg, France

The authors wish to make the following corrections to this paper [1]:

On the right panel of Figure 3, the second PCR illustration for PKR1 and PKR2 levels represents 48 h after MI, not 1 week after MI. We would like to change the labeling of these illustrations and add the comment in the figure legends that despite the increase in PKR1 and PKR2 levels, 48 h after the MI, PKR1 and PKR2 were almost depleted 1 week after MI.

The authors are sorry to report that in the Figure 3 right panel, the lower PCR illustrations are labelled as 1 week after MI, instead of 48 h after the MI. Since the expressions of these genes remain unchanged or even slightly reduced 1 week after MI, consequently the authors wish to make at this time the following corrections in Figure 3, the Figure 3 legend and related text in the manuscript.

These changes have no material impact on the conclusions of our paper. We apologize to our readers. We have recently been made aware of the error in the Figure 3 of our paper by Dr. Laurent Désaubry at the University of Strasbourg, INSERM UMR1260, Nanoregenerative medicine.

We would like to make the following corrections:

## 4. Prokineticin Signaling in Myocardial Infarction (MI)

The expression of prokineticins and their receptors is upregulated within 48 h after MI in mice (Figure 3A,B). Accordingly, Akt phosphorylation levels that are 30% higherin ischemic than in non-ischemic hearts indicate an activation of the compansatory cardioprotective signaling pathway, triggering the endogenous wound-healing process [38]. However, the PKR1 expression was reduced, while PKR2 expressions remain unaltered one week after MI (Figure 3C). Indeed, PK2 long form (PK2L) has not been detected in MI hearts one week after MI, (Figure 3C). Whether the alternative splicing of PK2L occurred during the heart remodeling after the MI needs to be studied.

In the mouse coronary ligation of MI model, the intra-cardiac PKR1 gene transfer utilizing adenovirus (Adv) carrying PKR1 cDNA (Adv-PKR1) induced a three-fold increase in the PKR1 level 24 h after MI, rising to a four-fold increase 48 h after MI, remaining elevated after 1 week after MI [38].

**Figure 3:**
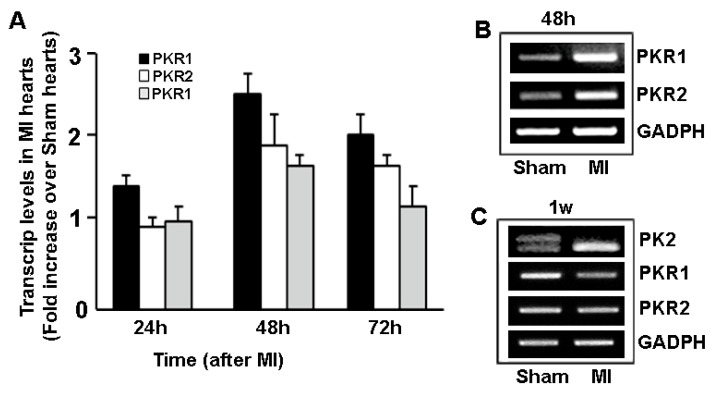
(**A**) Quantitative analyses of expression levels of prokineticin 2 (PK2) and its receptors, PKR1 and PKR2 in MI and sham operated mice hearts, 24, 48 and 72 hours after the operations. (**B**) Representative illustration of PCR analyses of the PKR1 and PKR2 transcripts 48h after MI. (**C**) Expression of transcripts of PK2 long form (PK2L, double bands) and short form (single bands), and PKRs in MI and sham operated hearts one week after MI.
